# Sero-prevalence and risk factors for hepatitis B virus infection among health care workers in a tertiary hospital in Uganda

**DOI:** 10.1186/1471-2334-10-191

**Published:** 2010-06-29

**Authors:** Abdhalah K Ziraba, Josephine Bwogi, Alice Namale, Caroline W Wainaina, Harriet Mayanja-Kizza

**Affiliations:** 1 African Population and Health Research Center, 2nd Floor Shelter Afrique Center, Longonot Road, Upper Hill, P. O. Box 10787, 00100, Nairobi Kenya; 2 Department of Epidemiology and Population Health, Centre for Population Studies, London School of Hygiene and Tropical Medicine. 49-51 Bedford Square, London, WC1B 3DP, UK; 3 Uganda Virus Research Institute, P. O. Box 49, Entebbe, Uganda; 4 Mulago Hospital, P. O. Box 7051 Kampala, Uganda; 5 Institute of Tropical Medicine and Infectious Diseases, P. O. Box 62000-00200, Nairobi, Kenya; 6 Makerere University, College of Health Sciences, P. O. Box 7072, Kampala, Uganda

## Abstract

**Background:**

Hepatitis B virus (HBV) infection is a global public health challenge. Prevalence of current hepatitis B virus infection in the general population in Uganda is about 10%. Health care workers (HCW) have an extra risk of getting infected from their workplace and yet they are not routinely vaccinated against HBV infection. This study aimed at estimating prevalence of hepatitis B virus infection and associated risk factors among health care workers in a tertiary hospital in Uganda.

**Methods:**

Data were obtained from a cross sectional survey conducted in Mulago, a national referral and teaching hospital in Uganda among health care workers in 2003. A proportionate to size random sample was drawn per health care worker category. A structured questionnaire was used to collect data on socio-demographic characteristics and risk factors. ELISA was used to test sera for HBsAg, anti-HBs and total anti-HBc. Descriptive and logistic regression models were used for analysis.

**Results:**

Among the 370 participants, the sero-prevalence of current hepatitis B virus infection was 8.1%; while prevalence of life time exposure to hepatitis B virus infection was 48.1%. Prevalence of needle stick injuries and exposure to mucous membranes was 67.8% and 41.0% respectively. Cuts were also common with 31.7% of doctors reporting a cut in a period of one year preceding the survey. Consistent use of gloves was reported by 55.4% of respondents. The laboratory technicians (18.0% of respondents) were the least likely to consistently use gloves. Only 6.2% of respondents were vaccinated against hepatitis B virus infection and 48.9% were susceptible and could potentially be protected through vaccination. Longer duration in service was associated with a lower risk of current infection (OR = 0.13; p value = 0.048). Being a nursing assistant (OR = 17.78; p value = 0.007) or a laboratory technician (OR = 12.23; p value = 0.009) were associated with a higher risk of current hepatitis B virus infection. Laboratory technicians (OR = 3.99; p value = 0.023) and individuals with no training in infection prevention in last five years (OR = 1.85; p value = 0.015) were more likely to have been exposed to hepatitis B virus infection before.

**Conclusions:**

The prevalence of current and life time exposure to hepatitis B virus infection was high. Exposure to potentially infectious body fluids was high and yet only a small percentage of HCW were vaccinated. There is need to vaccinate all health care workers as a matter of policy and ensure a safer work environment.

## Background

Globally there are about 360 million chronic carriers of hepatitis B virus and over one million people die each year as a result of acute fulminant liver disease or hepatitis B virus (HBV) induced cirrhosis and liver cancer [1]. The burden of hepatitis B virus infection is highest in the developing world particularly Asia and sub-Saharan Africa [2-4]. World Health Organization estimates that the prevalence of hepatitis B virus infection in Africa is on average more than 10% [5,6]. Recent studies carried out in Uganda showed that the prevalence of current hepatitis B virus infection in the general population is about 10% [7].

Although most infections in the developing world occur in childhood and early adulthood, a significant proportion of non-immune adults remain at risk. Hepatitis B virus infection is a recognized occupational hazard as non-immune health care workers (HCW) stand a risk of getting infected from their work place [8-11] . Generally HCW who perform invasive procedures for example surgeons, dentists, emergency workers and those who handle human specimens like the laboratory technicians have been consistently shown to have higher prevalence of hepatitis B virus infection than their counterparts [12-14]. The differences in HBV infection rates may reflect disparities in the risk of exposure to infection [14,15]. For instance one study conducted among dental students and dentists revealed that a significantly higher proportion of dentists tended to use gloves compared to the dental students [16], while another study showed that 38% of professional HCW were vaccinated compared to only 3.5% of the housekeeping staff in the same hospital [17].

Because available treatment for hepatitis B virus infection does not provide a complete cure, prevention remains crucial [18]. A safe, effective and highly acceptable HBV vaccine has been around since 1982 [19,20], but its use among HCW in the developing world is low [21-24]. Limited access to vaccination by HCW is a consequence of lack of initiative from governments to formulate policy and guidelines to ensure that all HCW get vaccinated.

Whereas the literature on hepatitis B virus infection in Uganda is growing, there is still paucity of information on HBV among HCW. This paper contributes to this discourse by presenting the prevalence estimates and risk factors for hepatitis B virus infection among health care workers. It also presents an assessment of availability of infection prevention strategies including vaccination.

## Methods

### Setting and study population

This study was conducted from March to April 2003 in Mulago Hospital, Uganda's largest national referral and teaching hospital, with a bed capacity of about 1500 beds. At the time of the study, the hospital had about 191 specialists, 125 medical officers, 80 intern doctors, 132 paramedical officers (clinical officers, ophthalmic officers, physiotherapists radiographers), 893 nurses (nurses, midwifes, and nursing assistants) and 88 laboratory technicians. Visiting practitioners, nursing and medical students were excluded from the study. Cleaners were also excluded because they were not employees of the hospital.

### Study design, sampling and participant recruitment

We used a cross sectional study design. Using a staffing list as the sampling frame, a proportionate random sample of health care workers was drawn by health care worker category (stratum) to participate in the survey. The strata were based on health care worker cadre thus; specialists, medical officers, paramedical officers, laboratory technicians, and nurses/midwives (nurses, midwives, nursing assistants and theatre attendants). The staffing list obtained from the hospital administration was cleaned excluding those who didn't meet the inclusion criteria. A very small number (about 20) of eligible and sampled individuals turned down participation. These were replaced with individuals randomly picked from the same stratum. The estimated sample size was 370 participants assuming a prevalence of HBsAg of 40%, one of the highest reported among laboratory technician category of HCW [25]. Sample size was derived using the following formula: (*N *= *Z*^*2*^_*α/2*_*PQ/d*^*2*^), where *N *= sample size; *Z*_*α/2 *_= standard normal distribution abscissa corresponding to 95% confidence interval (1.96); *P *= proportion of HBsAg reported in similar study noted above (40%); *Q = (1-P); *and *d *= desired level of precision (5%). The proposal was approved by the Makerere University Faculty of Medicine Research and Ethics Committee. Mulago Hospital Administration also gave approval for the study to be conducted on its staff. Written informed consent was obtained from each participant before any procedures were carried out.

### Measurements

A structured questionnaire was used to collect individual socio-demographic characteristics, work environment, history of exposure to patient body fluids, duration in service, use of protective wear, vaccination status, willingness to be vaccinated, perceptions on safety of equipment, and medical waste management in work stations. Perception of hepatitis B virus infection risk was assessed using a scale of low, moderate and high. The serologic markers tested were, hepatitis B surface antigen (HBsAg), Hepatitis B surface antibody (anti-HBs) and total hepatitis B core antibody (total anti-HBc). Presence of HBsAg in blood signifies acute or chronic persistent HBV infection. Anti-HBs are produced in response to HBsAg and confer immunity to re-infection and their presence indicates immunity to HBV infection following an infection or successful immunization with hepatitis B vaccine. Anti-HBc is directed against the core antigen following a natural infection and normally persists for life. Its presence may indicate a current or past resolved infection [26].

### Laboratory investigations

Using an aseptic technique, about 5 ml of blood were drawn from each participant and immediately put in a vacutainer containing a clot activator. The vacutainers were labeled indicating the serial number and date of sample collection. Blood samples were then taken to the laboratory in Nakasero Blood Bank for separating sera. Sera were stored at -20°C awaiting investigations. We used Enzyme Linked Immunosorbent Assay (ELISA), (murex version 3 manufactured by Murex Biotech limited) to carry out HBs Ag, anti-HBs and total anti-HBc tests. Results were determined spectrophotometrically and interpretation of test results was as per manufacturer's guidelines.

### Data analysis

Using unique identifiers, laboratory test results were linked to the questionnaire data. The health care worker categories were re-categorized based on similarities in their roles in the health facility and adequacy of numbers for example specialists and medical officers were grouped as "doctors", while the broad group of nurses/midwives/nursing assistants was split into the respective categories of nurse, midwife and nursing/theatre assistants at analysis.

Analysis was carried out using Stata Version 10 statistical software. For bivariate analysis, we computed odds ratios and confidence intervals using the trend chi squared test. Factors that were significantly associated to the outcomes at bivariate analysis at 10% level of significance and some of those that have been reported in the literature to be associated with hepatitis B virus infection were included in logistic regression models.

## Results

Out of the 370 participants, 73.5% were female and the mean age for all participants was 36.4 years (SD = 8.4). On average females were older than males (36.9 years compared to 34.6 years) respectively. Female HCW on average had spent about 14 years in service compared to about 8 years for the males. About 56.5% of HCW were working in a surgical department, 38.4% in a medical/pediatric department and only about 5.1% were working in laboratories.

### Potential for exposure at work place

Exposure to potentially infectious body fluids at the work place was assessed using a set of variables as shown in Table 1. Needle stick injuries in the last one year were reported in 67.8% of the respondents mainly affecting doctors, nurses, and laboratory technicians. The doctors were mainly affected during surgical procedures, while nurses were affected while preparing or giving medication and laboratory clinicians while recapping needles. Over 67.0% of all participants who reported a needle stick injury had been injured two or more times in the past one year (results not shown). Exposure of mucous membranes (mouth and eyes) to patient's body fluids occurred in about 41.0% of respondents in the previous year affecting mainly doctors and midwives. Over 65.0% of respondents thought that the work place and surfaces are not adequately disinfected mainly due to limited availability of disinfectants (results not shown). Doctors suffered most cuts with 31.7% of them having had a cut at least once in the last one year. Consistent use of gloves (that is, use of gloves each time they carried out a procedure involving body fluids) as a means of preventing risk of infection was reported in 55.4% of respondents. The laboratory technician category was the least likely to consistently use gloves (18.2%), while 87.0% of the nursing assistants reported consistent use of gloves. Although continuing medical education is mandatory for all HCW, training in infection control in the last five years was reported by only 34.3% of the respondents. Results in Table 1 also show that about 58.5% of respondents reported that their risk of getting infected was high compared to 13.9% who reported that it was low. Nursing assistants (73.9%) and laboratory technicians (77.3%) categories had the highest proportions of individuals who thought their own risk of infection was high.

**Table 1:** Potential for exposure to hepatitis B virus infection by socio-demographic characteristics

			Potential for exposure to infection (%)							
		
Characteristics	Number	Needle stick injury *(Yes)*	Cuts *(Yes)*	Muco-cutaneous exposure *(Yes) *	Consistent use of gloves *(Yes)*	Vaccinated *(Yes)*	Use other protective gear *(yes)*	Perceived risk of infection		
								
								Low	Moderate	High
Sex										
Female	272	68.8	20.6	41.3	55.2	4.4	9.6	13.4	25.4	61.2
Male	98	65.3	29.6	40.2	56.1	11.2	15.3	15.3	33.7	51.0
Age										
20-29 years	80	70.0	23.8	46.3	50.0	10.0	6.3	13.9	25.3	60.8
30-39 years	168	72.5	23.4	47.3	56.9	4.8	10.8	13.3	29.7	57.0
40-49 years	90	63.3	23.3	31.1	56.7	4.4	13.6	14.4	23.3	62.2
50+ years	32	53.1	18.8	25.0	59.4	9.4	18.8	16.1	35.5	48.4
Religion										
Catholics	99	66.7	20.2	29.3	55.6	5.1	13.1	14.4	29.9	55.7
Protestants	160	70.0	28.1	43.0	52.5	2.5	9.4	13.2	28.9	57.9
Moslems	27	59.3	25.9	48.2	63.0	3.7	7.7	3.7	11.1	85.2
Other Christians	84	67.9	15.5	48.8	58.3	15.5	13.1	18.1	27.7	54.2
Marital status										
Single	127	71.7	23.6	40.2	52.0	9.5	5.6	12.0	27.2	60.8
Married	219	66.2	22.4	43.3	57.5	5.0	13.3	15.1	28.0	56.9
Separated/widowed	24	62.5	25.0	25.0	54.2	0.0	20.8	13.0	26.1	60.9
Years in service										
< 10 years	158	69.0	24.1	44.9	53.8	9.5	9.5	14.7	31.4	53.9
10-19 years	119	73.1	20.2	44.4	60.5	2.5	10.3	11.9	22.0	66.1
20+ years	93	59.1	24.7	30.1	51.6	5.4	15.1	15.2	28.3	56.5
Cadre										
Doctor/specialist	79	72.2	31.7	57.7	51.9	20.3	16.5	15.6	35.1	49.4
Clinical/dental officer	25	36.0	28.0	20.0	64.0	0.0	16.0	44.0	20.0	36.0
Nurse	196	71.4	21.9	39.0	55.6	3.1	9.7	13.3	27.2	59.5
Midwife	25	80.0	12.0	64.0	60.0	0.0	0.0	8.3	20.8	70.8
Laboratory technician	22	72.7	18.2	18.2	18.2	4.6	4.8	0.0	22.7	77.3
Nursing assistant	23	39.1	13.0	21.7	87.0	0.0	17.4	0.0	26.1	73.9
Department										
Surgical departments	209	67.3	26.9	45.4	66.4	5.3	15.4	15.5	25.7	58.7
Laboratories	19	61.1	16.7	11.8	22.2	5.6	5.9	0.0	22.2	77.8
Medical/Paediatrics	142	69.5	18.4	39.0	44.0	7.8	5.0	13.0	30.9	56.1
Total	370	67.8	23.0	41.0	55.4	6.2	11.1	13.9	27.6	58.5

### Vaccination against Hepatitis B Virus Infection

Only 6.2% of respondents were vaccinated against hepatitis B virus infection. Doctors (20.3%), males (11.2%), other Christians (15.5%) and respondents who were younger than 30 years of age (10.0%) reported higher rates of vaccination than their counterparts. Of those who had ever been vaccinated, about 34.8% completed the recommended three dose schedule. The majority (65.2%) got vaccinated either through payment from own pocket, or through a promotional activity by a pharmaceutical company. Over 95% of health care workers were willing to be vaccinated if the vaccine was provided free of charge and about 65.6% were willing to be vaccinated if the cost of vaccination was subsidized to a lower fee.

From Table 2, it can be seen that 48.9% of all participants did not have any of the markers and thus classified as susceptible to infection. About 23.0% were positive for anti-HBs and anti-HBc, an indication that they were immune to HBV infection following a natural infection while only 3.0% were immune following vaccination. Current HBV infection (HBsAg positive and anti-HBc positive) was present in 8.1% of respondents. Overall, 17.0% of respondents were classified as indeterminate because they had a positive anti-HBc result and were negative for HBsAg and anti-HBs. Four possible interpretations are possible: resolving infection (window phase), remote resolved infection with low anti-HBs, chronic infection with low levels of HBsAg or false positive anti-HBc.

**Table 2:** Interpretation of serologic markers: HBV infection status and corresponding percentages

Serologic markers			Interpretation	N = 370 (%)
		
HBsAg	Anti-HBs	Anti-HBc		
Negative	Negative	Negative	Susceptible	181 (48.9%)
Negative	Positive	Positive	Immune after infection	85 (23.0%)
Negative	Positive	Negative	Immune after vaccination	11 (3.0%)
Positive	Negative	Positive	Current infection	30 (8.1%)
Negative	Negative	Positive	Indeterminate: *Four possibilities*	63 (17.0%)
			*i) Resolving infection (Window phase)*	
			*ii) Remote resolved infection with low anti-HBs*	
			*iii) Chronic infection with low levels of HBsAg*	
			*iv) False positive anti-HBc, hence susceptible.*	

### HBV infection status by socio-demographic characteristics

Table 3 shows prevalence of current HBV infection and life time exposure to HBV infection by socio-demographic characteristics. By HCW category, nursing assistants had the highest prevalence of current infection at (26.1%), followed by laboratory technicians (18.2%) and clinical/dental officers (12.0%). Individuals who had been in service for longer had lower prevalence of current infection, as were unmarried HCW. By department, participants from laboratory units had the highest prevalence of current infection of about 15.8%. About 48.1% (95% CI: 43.0-53.2) had evidence of life time exposure to HBV infection. Participants who were older, Muslims, widowed/separated, those who had been in service for longer, and those from surgical and laboratory departments had higher prevalence of exposure to HBV infection. Laboratory technicians had the highest prevalence of exposure to HBV infection with 72.7% being positive.

**Table 3:** Distribution of current HBV infection and lifetime exposure by socio-demographic characteristics

Characteristics	Total Number (N)	Current HBV infection n (%)	Life time exposure to HBV infection n (%)
Gender			
Female	272	21(7.7)	52(53.1)
Male	98	9(9.2)	126(46.3)
Age			
20-29 years	80	5(6.3)	36(45.0)
30-39 years	168	16(9.5)	80(47.6)
40+ years	122	9(7.4)	62(50.8)
Religion			
Catholics	99	11(11.1)	51(51.5)
Protestants	160	16(10.0)	76(47.5)
Moslems	27	2(7.4)	14(51.9)
Other Christians	84	1(1.2)	37(44.1)
Marital status			
Single	127	8(6.3)	57(44.9)
Married	219	19(8.7)	107(48.9)
Separated/widowed	24	3(12.5)	14(58.3)
Years in service			
< 10 years	158	14(8.9)	76(48.1)
10-19 years	119	10(8.4)	55(46.2)
20+ years	93	6(6.5)	47(50.5)
Cadre			
Doctor	79	3(3.8)	33(41.8)
Clinical/dental Officer	25	3(12.0)	12(48.0)
Nurse	196	13(6.6)	93(47.5)
Midwife	25	1(4.0)	11(44.0)
Laboratory technician	22	4(18.2)	16(72.7)
Nursing assistant	23	6(26.1)	13(56.5)
Department			
Surgical departments	209	17(8.1)	102(48.8)
Laboratories	19	3(15.8)	12(63.2)
Medical/Paediatrics	142	10(7.0)	64(45.1)
Total	370	30(8.1)	178(48.1)
		[95% CI; 5.3-10.9]	[95% CI; 43.0-53.2]

### Risk factors for current hepatitis B virus infection and life time exposure to hepatitis B virus infection

Table 4 shows unadjusted and adjusted odds ratios for risk factors for current hepatitis B virus infection. In the logistic regression model, a number of variables were found to be significantly associated with current hepatitis B virus infection at 5% level of significance (Table 4). The category of "other Christians (mainly Pentecostal church, and Seventh Adventists) were at a lower risk of hepatitis B virus infection compared to Catholics (OR = 0.07; P value = 0.017). The duration (number of years spent) in service was inversely associated with the risk of current hepatitis B virus infection. The higher the number of years spent in service the lower the risk of current infection. For example the odds of current hepatitis B virus infection in individuals who had spent 20 or more years compared to the odds of current hepatitis B virus infection in those who had fewer than ten years in service was 0.13 (P value = 0.048), while this ratio was 0.25 in those individuals who had spent between 10 and 20 years (P value = 0.039). Laboratory technicians and nursing assistants had about 12 times (OR = 12.23; P value = 0.009) and 18 (OR = 17.78; P value = 0.007) more risk than doctors respectively. Individuals who had a history of a cut, mucocutaneous exposure, surgical operation, and those who did not routinely use other protective gear (other than gloves) had a higher risk of infection although this didn't reach significance level.

**Table 4:** Risk factors: Results from a logistic regression model for current hepatitis B virus infection

Variables	Current Hepatitis B virus infection			
	
	Unadjusted OR [95% CI]	P-value	Adjusted OR [95% CI]	P-value
Gender(Ref = Male)				
Female	0.81[0.36-1.84]	0.620	1.09[0.29-4.11]	0.903
Age (Ref = 20-29 years)				
30-39 years	1.60[0.56-4.54]	0.377	2.84[0.74-10.85]	0.127
40+ years	1.24[0.40-3.86]	0.705	3.63[0.47-27.97]	0.215
Religion(Ref = Catholics)				
Protestants	0.88[0.39-1.98]	0.752	0.74[0.30-1.87]	0.530
Moslems	0.62[0.13-2.98]	0.549	0.29[0.05-1.85]	0.193
Other Christians	0.10[0.01-0.79]	0.029	0.07[0.01-0.61]	0.017
Marital status(Ref = Single)				
Married	1.43[0.61-3.38]	0.409	1.39[0.49-3.96]	0.539
Separated/widow	2.07[0.51-8.45]	0.310	1.29[0.20-8.43]	0.788
Years in service(Ref = < 10 years)				
10-19 years	0.94[0.40-2.21]	0.893	0.25[0.07-0.93]	0.039
20+ years	0.72[0.27-1.95]	0.522	0.13[0.02-0.99]	0.048
Cadre(Ref = Doctors)				
Clinical/dental Officer	3.27[0.62-7.39]	0.164	4.51[0.59-34.65]	0.147
Nurse	1.77[0.49-6.41]	0.383	2.69[0.51-14.13]	0.242
Midwife	1.00[0.10-10.07]	1.000	2.09[0.13-33.42]	0.602
Laboratory technician	5.33[1.09-5.99]	0.038	12.23[1.85-81.08]	0.009
Nursing assistant	8.47[1.92-7.33]	0.005	17.78[2.17-45.87]	0.007
Changed department (Ref = Yes)				
No	0.77[0.36-1.64]	0.495	0.59[0.21-1.62]	0.307
Needle stick injury(Ref = Yes)				
No	1.63[0.76-3.48]	0.206	1.76[0.68-4.51]	0.242
Cut injury(Ref = Yes)				
No	0.68[0.30-1.54]	0.353	0.60[0.21-1.66]	0.324
Mucocutaneous exposure(Ref = Yes)				
No	1.17[0.54-2.54]	0.692	0.64[0.24-1.70]	0.369
Use gloves (Ref = Always)				
Some times	0.82[0.38-1.75]	0.599	0.89[0.37-2.16]	0.795
Vaccinated (Ref = Yes)				
No	1.68[0.22-3.03]	0.620	0.42[0.04-4.22]	0.461
Disinfection (Ref = Adequate)				
Not adequate	0.56[0.25-1.29]	0.174	0.65[0.25-1.72]	0.390
Don't know	0.54[0.14-2.08]	0.370	0.61[0.13-2.75]	0.518
Surgical operation (Ref = Yes)				
No	0.74[0.35-1.58]	0.437	0.53[0.21-1.32]	0.173
Blood transfusion (Ref = Yes)				
No	1.10[0.25-4.91]	0.899	1.31[0.22-7.59]	0.767
Risk perception (Ref = Low)				
Moderate	1.22[0.36-4.16]	0.756	0.73[0.17-3.12]	0.674
High	1.05[0.34-3.25]	0.938	0.69[0.18-2.66]	0.585
Trained in infection prevention(Ref = Yes)				
No	0.89[0.41-1.94]	0.772	0.75[0.30-1.88]	0.533
Body scarification(Ref = Yes)				
No	0.94[0.44-2.02]	0.880	1.03[0.39-2.69]	0.951

With regard to life time exposure to hepatitis B virus infection (Table 5), being a laboratory technician was associated with about 4 times risk of one having ever been exposed to hepatitis B virus infection (OR = 3.99; P value = 0.023) compared to doctors. Individuals with no history of surgical operation were at a higher risk of exposure (OR = 1.78; P value = 0.028). Having no training in infection prevention was associated with a 2 times (OR = 1.85; P value = 0.015) higher risk of having been exposed to hepatitis B virus infection. The rest of the other variables were not significantly associated with exposure to hepatitis B virus infection in the regression model.

**Table 5:** Risk factors: Results from a logistic regression model for life time exposure to Hepatitis B Virus infection

Variables	Life time exposure to HBV infection			
	
	Unadjusted OR [95% CI]	P-value	Adjusted OR [95% CI]	P-value
Gender(Ref = Male)				
Female	0.66[0.41-1.07]	0.090	0.57[0.28-1.18]	0.129
Age (Ref = 20-29 years)				
30-39 years	1.04[0.60-1.78]	0.899	1.05[0.53-2.10]	0.889
40+ years	1.32[0.74-2.35]	0.339	1.54[0.51-4.60]	0.443
Religion(Ref = Catholics)				
Protestants	0.87[0.52-1.44]	0.580	0.93[0.53-1.63]	0.804
Moslems	1.22[0.52-2.87]	0.648	1.69[0.65-4.37]	0.279
Other Christians	1.00[0.55-1.81]	0.996	1.05[0.54-2.03]	0.892
Marital status(Ref = Single)				
Married	1.10[0.71-1.72]	0.667	1.31[0.78-2.20]	0.315
Separated/widow	1.53[0.64-3.69]	0.341	2.20[0.79-6.14]	0.133
Years in service(Ref = < 10 years)				
10-19 years	0.85[0.53-1.39]	0.527	1.09[0.55-2.19]	0.800
20+ years	1.14[0.68-1.93]	0.616	1.02[0.35-2.96]	0.966
Cadre(Ref = Doctors)				
Clinical/dental Officer	0.72[0.28-1.82]	0.484	0.57[0.19-1.68]	0.310
Nurse	1.09[0.63-1.86]	0.765	1.41[0.66-3.01]	0.379
Midwife	0.85[0.34-2.13]	0.727	0.96[0.31-2.99]	0.944
Laboratory technician	3.39[1.20-9.63]	0.022	3.99[1.21-13.19]	0.023
Nursing assistant	0.82[0.32-2.12]	0.680	0.72[0.22-2.36]	0.591
Changed department (Ref = Yes)				
No	1.35[0.89-2.05]	0.159	1.33[0.81-2.18]	0.253
Needle stick injury(Ref = Yes)				
No	1.11[0.72-1.73]	0.637	1.02[0.61-1.70]	0.950
Cut injury(Ref = Yes)				
No	1.56[0.94-2.58]	0.083	1.56[0.88-2.74]	0.127
Mucocutaneous exposure(Ref = Yes)				
No	1.08[0.71-1.66]	0.714	0.90[0.55-1.49]	0.694
Use gloves (Ref = Always)				
Some times	1.04[0.69-1.58]	0.847	1.02[0.63-1.64]	0.944
Vaccinated (Ref = Yes)				
No	0.60[0.23-1.52]	0.277	0.56[0.20-1.61]	0.286
Protective wear always available (Ref = Yes)				
No	0.97[0.50-1.86]	0.926	1.04[0.51-2.14]	0.910
Surgical operation (Ref = Yes)				
No	1.57[1.02-2.44]	0.042	1.78[1.06-2.97]	0.028
Blood transfusion (Ref = Yes)				
No	0.98[0.44-2.18]	0.960	0.64[0.26-1.60]	0.341
Trained in infection prevention(Ref = Yes)				
No	1.67[1.07-2.61]	0.023	1.85[1.12-3.03]	0.015
Body scarification(Ref = Yes)				
No	1.02[0.67-1.57]	0.914	0.89[0.54-1.48]	0.664

## Discussion

This study shows that hepatitis B virus sero-markers are prevalent among health care workers in a Ugandan tertiary hospital. The prevalence of current infection was 8.1% and that for life time exposure was 48.1%. Findings from a similar study conducted among health care workers in Uganda found a comparable prevalence of 9.0% for current infection, however that for life time exposure to hepatitis B virus infection was much higher at 60.1% [24]. Our results show marked variations in prevalence of Hepatitis B sero-markers by type of health care worker, department, and religion. Similar observations have been reported in other studies with laboratory technicians, dentists and nurses being disproportionately affected [12,27]. The variations among the different cadres of HCW might be a reflection of the different levels of risk of exposure to a hazardous work environment the different categories of health care workers operate in. However, other risks such as sexual activity that were not explored in this study could explain the variations. Differentials in knowledge about the dangers of hepatitis B virus infection and the available prevention strategies might also partly explain the observed differences in prevalence. Indeed our results show that individuals with no training in infection prevention had a higher risk of life time exposure to hepatitis B virus infection. Nationally, the prevalence of HBsAg and anti-HBc shows great variation along demographic lines such as ethnicity, region, age, religion, among others which may influence the distribution of infection seen among the HCW. For example a population based survey revealed that the prevalence of anti-HBc among the Banyankole tribe in the western part of the country was 28.3% while that of Karimojongs in the north eastern part of the country was 93.2%. In the same study, the prevalence of HBsAg among Catholics was 11.5% compared to 7.1% among Moslems and 2.6% among other Christians (other than Catholics and Protestants) [6].

Exposure to potentially infected patient fluids is quite high. We found a considerably high proportion of individuals who had had either a needle stick injury, cut or other form of exposure and yet only 6.2% were vaccinated with just about a third of these getting the recommended three doses. The incomplete vaccination schedules might explain the observation that while 6.2% of all participants reported that they had ever been vaccinated, only 3% of participants were immune as a result of vaccination. Although several studies including this one have shown that individuals from certain departments tend to have a higher prevalence of hepatitis B, our results further show that exposure to potentially infectious body fluids particularly through needle stick injuries is high across the board. Given the high efficiency of transmission of hepatitis B virus and the frequent use of injections, most health care workers even those in non-surgical departments are at a considerably high risk.

Although 48.1% of all participants had ever been exposed to hepatitis B virus infection and some of them got immune after the infection, a sizeable 48.9% remained susceptible to infection and could potentially benefit from vaccination. The 6.2% vaccination coverage reported in this study is very low compared to other developing countries like Pakistan with vaccination coverage of over 80% [28]. Vaccination of health care workers against Hepatitis B virus in Uganda is not mandatory and there is no formal framework for delivering vaccines to HCW. Results show that a large proportion of HCW appreciate the need to be vaccinated, with more than 95% indicating that they were willing to be vaccinated if the vaccine was provided free of charge. The lack of policy and a formal delivery system targeting HCW is a key challenge.

There is a complex multiplicity of risks of exposure and it is difficult to tease out the biggest contributors to infection. For example whereas the laboratory technician category had one of the highest proportion of HCW who thought that their own risk of infection was high, they had one of the lowest rates of consistent use of gloves. Doctors who were on average at high risk of getting a needle stick injury (NSI) or cut were more likely to be vaccinated potentially canceling out the excess risk. Also the risk of childhood and sexually acquired infections was not accounted for in this study thus making attribution of excess risk to occupational exposure a bit challenging. After controlling for other variables, longer duration in service remained significantly associated with a lower risk of current infection. This finding is at variance with what has been reported in other studies where prevalence of HBsAg was highest amongst the longest serving participants [24,29]. Being a laboratory technician or a nursing assistant was independently associated with a higher risk of current hepatitis B virus infection. This finding is in line with other studies [12]. Nursing assistants are at the bottom of the nursing hierarchy and are also closer to patients on a routine basis. Unlike their senior counterparts, they receive no formal training but rather learn on the job. These differences might explain the higher risk of infection in this group. Lack of in-service training on infection prevention was also associated with increased risk of life time exposure to infection. Several other factors such as history of blood transfusion, needle stick injuries, and vaccination status that have been reported in other studies to be associated with an increased risk were found not to be significantly associated with risk of hepatitis B virus infection in this study. With the advent of rigorous screening of blood for transfusion in Uganda it is likely that the risk of infection through transfusion is very minimal. On the other hand, needle stick injures and cuts are so prevalent as shown in this and other studies[14] that most health care workers have had an experience at least once during service.

## Limitations

The stratified random sampling approach used in this study meant that participation was proportionate to population size in a given stratum (HCW category). Some categories of HCW generally have few staff such as the dental and laboratory categories. Because of this the numbers in some cells were too small to allow for stable estimates to be derived (wide confidence intervals). In future similar studies should consider the need to oversample in underrepresented subgroups that have been reported to have a high prevalence. Additionally other factors known to contribute to the risk of infection such as sexual behavior were not assessed. This is one area that might need consideration while designing data collection tools for similar studies. A set of questions akin to those administered to HIV/AIDS sexual behavior studies could be considered. Recall bias is a potential source of bias in this study as respondents may not be able to accurately remember events that happened long time ago. Lastly these results might have limited generalizibility given that participants were drawn from a single large teaching hospital. The situation in smaller health care facilities might be different.

## Conclusions

The prevalence of current hepatitis B virus infection and life time exposure to hepatitis B virus infection among health care workers was high. Exposure to potentially infectious body fluids was also high and yet only a small percentage of HCW are vaccinated against hepatitis B virus infection. Considering the risk of liver cirrhosis, hepatocellular carcinoma and transmission of HBV to patients, there is need to focus efforts on mitigating transmission through improving the work place environment and making use of the available vaccine by vaccinating all health care workers who are susceptible. Also given that the government of Uganda recognized the importance of hepatitis B virus infections among children and introduced vaccination in the childhood schedule, subgroups of adults at high risk such as HCW should be considered as a matter of policy. For example vaccination against hepatitis B virus infection could be made mandatory for preclinical medical and nursing students. The Ministry of Health could consider offering subsidized or free Hepatitis B vaccination to HCW. In addition education on infection control and other strategies for infection control need to be strengthened.

## Competing interests

The authors declare that they have no competing interests.

## Authors' contributions

AKZ conceived and developed the idea, spearheaded proposal development, data collection supervision, data management and analysis. He also took lead role in writing up the manuscript. JB participated in proposal development, data collection supervision and manuscript preparation. CWW participated in data analysis, literature review and manuscript writing. HMK and AN participated in developing the project protocol, interpretation of results, overall supervision and manuscript preparation. All authors read and approved the final manuscript.

## Pre-publication history

The pre-publication history for this paper can be accessed here:

http://www.biomedcentral.com/1471-2334/10/191/prepub
